# Transcriptomic Changes of Telencephalon and Hypothalamus in Largemouth Bass (*Micropterus salmoides*) Under Crowding Stress

**DOI:** 10.3390/biology14070809

**Published:** 2025-07-03

**Authors:** Meijia Li, Leshan Yang, Ying Liu

**Affiliations:** 1 College of Biosystems Engineering and Food Science (BEFS), Zhejiang University, Hangzhou 310058, China; 2 Key Laboratory of Environment Controlled Aquaculture, Ministry of Education, Dalian 116023, China; y2035864420@gmail.com

**Keywords:** crowding stress, brain transcriptome, largemouth bass, neuroinflammation, cell fate, synaptic plasticity

## Abstract

Crowding stress is an inevitable stressor in intensive farming, yet its underlying mechanisms are still obscure, severely hindering the aquaculture industry’s healthy development. As the primary sensory and regulatory organ for stressors, the brain plays a crucial role in stress responses. In this study, the effect of crowding stress on the telencephalon (Tel) and hypothalamus (Hy) has been explored using RNA sequencing. After four weeks of crowding stress, neuroinflammation-related genes were significantly induced in both the Tel and Hy. Additionally, cell fate-related processes were markedly altered. Neurogenesis-related pathways, including the *Wnt* and *Hedgehog* signaling pathways, were significantly enriched in both regions. The apoptosis-related genes (*caspase3*, *p53*) were predominantly downregulated in the Tel (log_2_Fold Change: −1.27 and −0.71, respectively), while ferroptosis-related genes (*ho1*, *ncoa4*) were specifically activated in the Hy (log_2_Fold Change: 1.15 and 0.73, respectively). The synaptic plasticity-related genes (*prkcg*, *cacna1d*) were significantly downregulated in both the Tel (log_2_Fold Change: −1.78 and −0.88) and Hy (log_2_Fold Change: −1.99 and −1.52). Furthermore, neurotransmitter synthesis (γ-aminobutyric acid (GABA) and serotonin (5-HT)) was disrupted in the Tel, whereas growth-related hormone gene expression was markedly altered in the Hy. These findings provide novel insights into the neurobiological mechanisms of chronic crowding stress in fish, laying a foundation for developing brain-targeted strategies to enhance welfare and mitigate stress in intensive largemouth bass farming.

## Simple Summary

Along with the rapid development of high-density recirculating aquaculture, intensive industrial farming has become widespread in the aquaculture industry. However, chronic crowding stress induced by intensive industrial farming can lead to delayed fish growth, suppressed immunity, and abnormal behavior. Although numerous studies have explored the causes of negative effects induced by crowding stress, the role of the brain, which is the primary sensory and central regulatory organ for stressors, has been neglected. In this study, the whole-transcriptome RNA expression profiles in the telencephalon and hypothalamus of largemouth bass have been explored under chronic crowding stress, providing new insights into the brain response mechanisms under crowding stress. Moreover, these findings facilitate the development of neural-based mitigation strategies.

## 1. Introduction

Crowding stress, an inevitable stressor in intensive farming, negatively affects fish growth, immunity, and behavior. For example, eight weeks of crowding stress significantly reduced the growth performance of the olive flounder (*Paralichthys olivaceus*) [1]. After 120 days of crowding stress, grass carp (*Ctenopharyngodon idella*) exhibited a chronic inflammatory response and reduced antioxidant enzymatic activity [2]. Nile tilapia (*Oreochromis niloticus*) showed significant declines in locomotor and exploratory behaviors following 6 weeks of crowding stress [3]. These stress responses align with Pickering’s three-stage model of fish stress [4]. The primary response is first triggered and involves changes in neuroendocrine activity. The secondary response manifests as a series of physiological, biochemical, and immune parameter alterations in the liver, spleen, and peripheral tissues due to neuroendocrine shifts. The tertiary response refers to phenotypic abnormalities in growth, immunity, and behavior resulting from the secondary physiological alterations [5]. Despite growing research on crowding stress in recent decades, its underlying mechanisms remain unclear. While most studies have focused on peripheral tissues (e.g., head kidney, liver, and skeletal muscle) linked to immunity and growth [6,7,8], the effects of crowding stress on the central nervous system remain poorly characterized, despite their critical role in initiating secondary and tertiary stress responses.

The fish brain serves as the central regulator of the stress response process, integrating sensory inputs to coordinate the body’s physiological response [9]. Emerging research has elucidated the region-specific effects of crowding stress on the brain, particularly in the hypothalamus and telencephalon. During stress exposure in the teleosts, activation of the hypothalamus–pituitary–interrenal (HPI) axis—the primary neuroendocrine pathway originating in the hypothalamus—induces a rapid release of corticotropin-releasing hormone (CRH) from hypothalamic neurons. The CRH further stimulates pituitary secretion of adrenocorticotropic hormone (ACTH), which subsequently acts on interrenal tissue to promote cortisol production [10]. For instance, in the Spotted goby (*Kryptolebias marmoratus*), 14 days of crowding stress significantly increased the number of CRH-positive neurons in the hypothalamic lateral tuberal nucleus [11]. Similarly, Nile tilapia (*Oreochromis niloticus*) reared under high-density conditions for 10 weeks exhibited an altered expression of HPI axis-related genes in the hypothalamus [12]. Crowding-induced HPI axis activation is modulated by neurotransmitters. In Gulf toadfish (*Opsanus beta*), chronic crowding stress was found to activate the HPI axis via regulation by brain 5-hydroxytryptamine 1A receptors [13]. The activation of the HPI axis involves complex positive and negative feedback loops between the neuroendocrine system and peripheral tissues, mediating adaptive physiological responses to stress, such as energy redistribution, immunity resistance, and growth inhibition [14]. However, HPI axis desensitization has been observed in some teleosts under chronic stress. For instance, Gilthead seabream (*Sparus aurata*) exposed to 45 days of crowding stress showed HPI axis unresponsiveness [15]. These findings underscore the necessity for further investigation into the relationship between chronic crowding stress and hypothalamic function. The telencephalon, a brain region critical for cognitive function, also responds to crowding stress. In rainbow trout (*Oncorhynchus mykiss*), 28 days of high-density farming significantly lowered telencephalic serotonin levels compared to low-density groups [16]. Moreover, neural plasticity, a crucial mechanism underlying learning, memory, and behavioral flexibility, is modulated by stress [17]. Adaptive stress (eustress), such as swimming exercise, has been shown to enhance neuroplasticity in the telencephalon of juvenile Atlantic salmon (*Salmo salar*) [18]. In contrast, excessive stress (distress), like prolonged cold exposure, impairs neural plasticity [19]. Nevertheless, the effects of crowding stress on neural plasticity in teleosts remain poorly understood.

Largemouth bass (*Micropterus salmoides*), which is a species highly favored by consumers globally (particularly in China), has experienced a steady rise in production over recent years. However, the expansion of intensive aquaculture systems has concurrently exacerbated crowding stress concerns [20,21]. Therefore, elucidating the mechanisms underlying crowding stress is essential for the healthy aquaculture of largemouth bass. Currently, numerous studies have characterized peripheral stress responses (e.g., oxidative activity in the head kidney and liver, metabolic states in the skeletal muscle) to crowding stress in largemouth bass [22,23,24]. However, a comprehensive understanding of brain responses remains limited, despite the brain’s critical role in coordinating stress adaptation. Whole-transcriptome sequencing has emerged as a powerful tool for characterizing genome-wide expression profiles and associated molecular pathways under stress conditions [25]. In our recent study, we have investigated the brain anatomy and function of largemouth bass, identifying the telencephalon and hypothalamus, rather than other brain regions such as the optic tectum, cerebellum, and medulla oblongata, as key regions closely related to cognitive function and growth regulation, respectively [26]. To elucidate the underlying mechanism of crowding stress on largemouth bass behavior and growth, transcriptomic sequencing, specifically targeting the telencephalon and hypothalamus, has been conducted. The aim is to uncover the effects of chronic crowding stress on brain health and function in largemouth bass, as well as to explore the underlying regulatory mechanisms related to fish behavior and growth, which may contribute to the development of novel management strategies for alleviating the detrimental effects induced by crowding stress in high-density farming environments.

## 2. Materials and Methods

### 2.1. Animals and Care

Adult largemouth bass (*Micropterus salmoides*) (mean body weight: 490.0 ± 15.0 g) were obtained from Dalian Ocean University, China. The fish were acclimated in 1000-L circular tanks (1.2 m diameter × 0.9 m height) at the Key Laboratory of Environment Controlled Aquaculture, Dalian Ocean University. The environmental parameters were maintained as follows: water temperature 20.0 ± 1.0 °C, dissolved oxygen ≥ 6.0 mg/L, pH 7.7, and total ammonia nitrogen < 0.3 mg/L. A 12:12 h light–dark photoperiod was implemented throughout the experiment. The stocking density was maintained at 10 kg/m^3^, and the fish were fed commercial feed (Tongwei, Chengdu, Sichuan, China) twice daily until satiation. A water exchange of two-thirds was performed in each tank for one-hour post-feeding to maintain water quality.

### 2.2. Treatment and Sample Collection

Thirty adult *M*. *salmoides* were randomly allocated into two experimental groups: a low-density group (10 kg/m^3^) and a high-density group (20 kg/m^3^). After four weeks of cultivation, as in previous studies [16,24,27], brain tissues were collected from each group for RNA sequencing experiments [20]. Specifically, after fasting for 24 h, each fish was randomly netted, anesthetized by exposure to liquid nitrogen for 20 s according to previous studies [28,29], and then dissected under aseptic conditions. Following dissection of the skull, the telencephalon and hypothalamus were carefully isolated and frozen in liquid nitrogen. For RNA sequencing, brain tissues from three individual fish were pooled to constitute one biological replicate, with three replicates in each experimental group. All samples were stored at −80 °C for subsequent analysis [26].

### 2.3. RNA Extraction and Library Preparation

RNA extraction was performed from the telencephalon and hypothalamus of largemouth bass via the TRIzol extraction method [30]. The mRNA was subsequently isolated from total RNA using poly(T)-conjugated magnetic beads. Following fragmentation, these fragmented mRNA molecules were utilized for cDNA synthesis [31]. Following cDNA synthesis, the fragmented cDNA underwent end repair and adaptor ligation. Using the AMPure XP system (Beckman Coulter, Brea, CA, USA), cDNA fragments with lengths in the range of 370–420 bp were selected. Next, PCR amplification was performed using Phusion High-Fidelity DNA polymerase (New England Biolabs, Ipswich, MA, USA). The resulting PCR products were purified to construct a sequencing library. The quality of the constructed library was assessed using the Agilent Bioanalyzer 2100 system (Agilent Technologies, Santa Clara, CA, USA), and the library was then sequenced on an Illumina Novaseq platform [32].

### 2.4. Differentially Expressed Genes Analysis and KEGG/GO Enrichment

The transcriptome sequencing data were analyzed following our previous study [20]. Briefly, raw data were processed into clean data, which were then aligned to the *M*. *salmoides* reference genome, and the fragments per kilobase per million (FPKM) value for each gene was calculated. Next, differentially expressed genes (DEGs) in the brain between high- and low-density groups were analyzed using the DESeq2 R package (version 1.20.0). Specifically, the *p*-value was used as the threshold for the differential gene expression test [33]. Functional enrichment analyses, including Kyoto encyclopedia of genes and genomes (KEGG) pathway and Gene ontology (GO) term analyses, were performed using the ClusterProfiler R package (version 3.18.0), with the pathways/terms showing a *p*-value < 0.05 and considered significantly enriched [34]. Principal component analysis (PCA) and a heatmap were carried out using NovoMagic Cloud Platform (http://magic.novogene.com) [35].

### 2.5. qPCR Validation of Selected Differentially Expressed Genes

To validate the RNA-seq results, a quantitative real-time PCR (qPCR) was carried out using the same RNA samples as those for RNA-seq library construction. Gene-specific primers for six target genes were listed in Table A2. qPCR amplification was conducted on a LightCycler^®^96 System (Roche, Basel, Switzerland) with SYBR Green I Master mix (Accurate Biology, Changsha, Hunan, China) [20]. Post-amplification dissociation curve analysis confirmed reaction specificity. Relative gene expression levels were calculated using the 2^−∆∆Ct^ method, with β-actin serving as the endogenous control [36].

## 3. Results

### 3.1. The Overall Characterization of the Transcriptome Data

**Figure 1:**
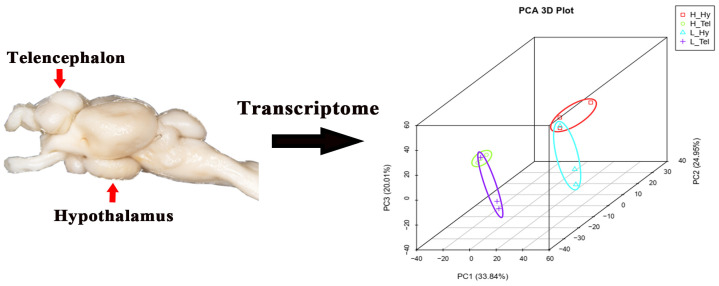
Principal component analysis (PCA) of transcriptomes from the telencephalon and hypothalamus of largemouth bass after crowding stress. H_Hy, high-density hypothalamus. H_Tel, high-density telencephalon. L_Hy, low-density hypothalamus. L_Tel, low-density telencephalon. The contribution ratios of the principal components (PC1, PC2, PC3) are displayed as percentage variance on the axis labels. Three biological replicates of each sample were grouped together and represented by one ellipse. Different-colored ellipses represent different groups.

A total of 260.47 and 257.21 million clean reads were obtained in the telencephalon and hypothalamus transcriptomes, respectively (Table A1). Following alignment to the *M*. *salmoides* reference genome, the clean reads showed high unique mapping rates ranging from 90.34% to 91.62%. To evaluate the reproducibility among biological replicates, principal component analysis (PCA) clustering was performed on four groups of data (including 12 samples) from the telencephalon (H_Tel) and hypothalamus (H_Hy) of the high-density group, as well as from the telencephalon (L_Tel) and hypothalamus (L_Hy) of the low-density group. The results demonstrated that the three samples within each experimental group clustered together, indicating high intra-group reproducibility. Additionally, samples from the two brain regions, telencephalon (H_Tel and L_Tel) and hypothalamus (H_Hy and L_Hy), each formed a separate cluster. Furthermore, there was a clear separation in clustering between the samples from the H_Tel and L_Tel groups, as well as between the samples from the H_Hy and L_Hy groups, suggesting that high-density treatment altered gene expression profiles in both the telencephalon and hypothalamus (Figure 1). Genome annotation revealed 30,394 expressed genes (FPKM > 0; 79.71% genome coverage), with 14,472 genes (37.95%) maintaining FPKM ≥ 1 across all groups.

### 3.2. Functional Classification of DEGs in the Telencephalon of Largemouth Bass Under Crowding Stress

**Figure 2:**
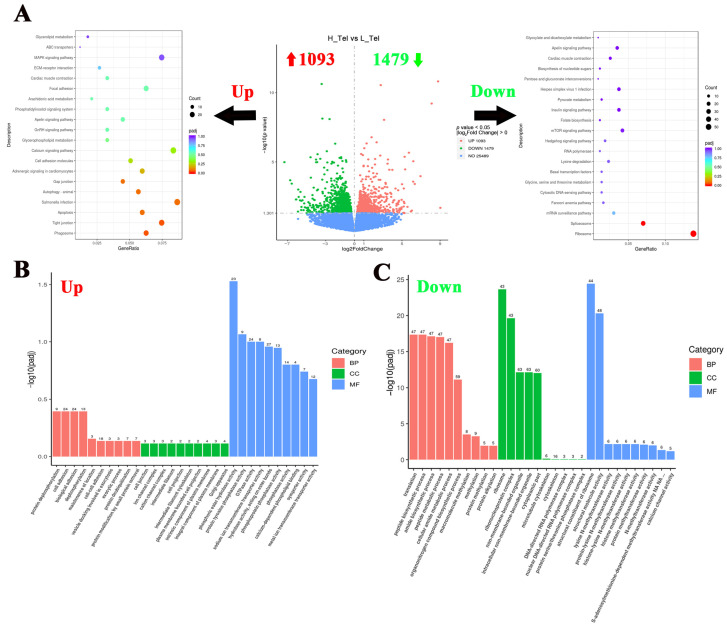
Functional classification of differentially expressed genes in the telencephalon of largemouth bass after crowding stress. (**A**) Volcano plot of differentially expressed genes and top 20 KEGG pathways enrichment based on upregulated/downregulated differential-expressed genes in the telencephalon between low- and high-density groups; (**B**) gene ontology (GO) enrichment analysis based on upregulated differential-expressed genes in the telencephalon between low- and high-density groups; (**C**) gene ontology (GO) enrichment analysis based on downregulated differential-expressed genes in the telencephalon between low- and high-density groups. Up, up-regulated differentially expressed genes (labeled with red upward arrow). Down, down-regulated differentially expressed genes (labeled with green downward arrow). BP, biological process. CC, cellular component. MF, molecular function. Top 10 GO terms in each of three groups have been listed in (**B**,**C**).

To investigate the crowding stress effects on the largemouth bass telencephalon, differentially expressed genes (DGEs) were screened using a significance threshold of *p <* 0.05. Comparative analysis between high- and low-density groups revealed 2572 significant DGEs in the telencephalon, comprising 1093 up-regulated and 1479 down-regulated genes (Figure 2A). To elucidate the impact of crowding stress on the telencephalic biological process, KEGG and GO enrichment analyses of DEGs were conducted. The KEGG pathway analysis revealed significant enrichment of up-regulated genes in the “Phagosome”, “Tight junction”, and “Apoptosis” pathways. Conversely, the down-regulated genes shown were significantly enriched in the “Ribosome” and “Spliceosome” pathways (Figure 2A). Regarding GO enrichment analysis, protein dephosphorylation and cell adhesion-related biological processes were significantly upregulated in the telencephalon, such as “protein dephosphorylation”, “cell adhesion”, and “phosphatase activity” GO terms. While metabolism and protein methylation processes were significantly down-regulated, such as “peptide metabolic process”, “methylation”, and “histone methyltransferase activity” (Figure 2B,C). The top 10 most significantly up-regulated and down-regulated genes (FPKM ≥ 1) in the telencephalon, ranked by log_2_Fold Change value (descending order), are listed in Table 1. The top 10 up-regulated genes included cytosolic non-specific dipeptidase, retinol-binding protein 4A, cathepsin D, etc. And the top 10 down-regulated genes included DNA-binding death effector domain-containing protein 2, Ras-related protein 7, protein kinase C gamma, etc.

### 3.3. Functional Classification of DEGs in the Hypothalamus of Largemouth Bass Under Crowding Stress

**Figure 3:**
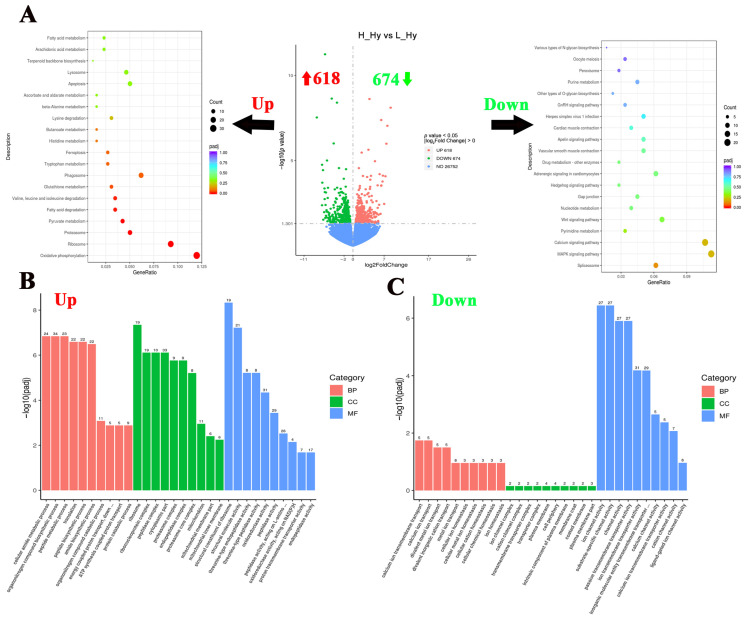
Functional classification of differentially expressed genes in the hypothalamus of largemouth bass after crowding stress. (**A**) Volcano plot of differentially expressed genes and top 20 KEGG pathways enrichment based on up-regulated/down-regulated differential-expressed genes in the hypothalamus between low- and high-density groups; (**B**) gene ontology (GO) enrichment analysis based on upregulated differential-expressed genes in the hypothalamus between low- and high-density groups; (**C**) gene ontology (GO) enrichment analysis based on downregulated differential-expressed genes in the hypothalamus between low- and high-density groups. Up, up-regulated differentially expressed genes (labeled with red upward arrow). Down, down-regulated differentially expressed genes (labeled with green downward arrow). BP, biological process. CC, cellular component. MF, molecular function. Top 10 GO terms in each of three groups have been listed in (**B**,**C**).

To investigate the hypothalamic responses to crowding stress in largemouth bass, 1292 DGEs were identified between the high- and low-density groups, comprising 618 up-regulated and 674 down-regulated genes (Figure 3A). In order to reveal the hypothalamic responses to crowding stress, a functional enrichment analysis of the DEGs has been conducted using the KEGG and GO databases. The KEGG pathway analysis revealed a significant enrichment of up-regulated genes in metabolic processes, including “Oxidative phosphorylation”, “Pyruvate metabolism”, “Fatty acid degradation”, and “Tryptophan metabolism” pathways. Conversely, the down-regulated genes were predominantly associated with cell fate-related pathways, particularly the “MAPK signaling pathway”, “Calcium signaling pathway”, “Wnt signaling pathway”, and “Hedgehog signaling pathway” (Figure 3A). Regarding GO enrichment analysis, peptide metabolism and biosynthesis-related processes were significantly upregulated in the hypothalamus, such as “peptide metabolic process”, “peptide biosynthetic process”, and “peptidase activity” GO terms. While ion channel activity and ion homeostasis-related processes were significantly down-regulated, such as “ion channel activity”, “channel activity”, and “metal ion transport” GO terms (Figure 3B,C). Based on the log_2_Fold Change value (descending order), the top 10 most significantly up-regulated and down-regulated genes (FPKM ≥ 1) in response to crowding stress were presented in Table 2. The highest up-regulated transcripts included D-dopachrome decarboxylase-A-like, interferon alpha-inducible protein 27, and somatostatin 1. Conversely, the most down-regulated genes comprised thyroid-stimulating hormone subunit beta, polyunsaturated fatty acid lipoxygenase ALOX15B, and NAC1_CANLF sodium/calcium exchanger 1.

### 3.4. Impact of Crowding Stress on Inflammation and Cell Fate in the Telencephalon and Hypothalamus of Largemouth Bass

**Figure 4:**
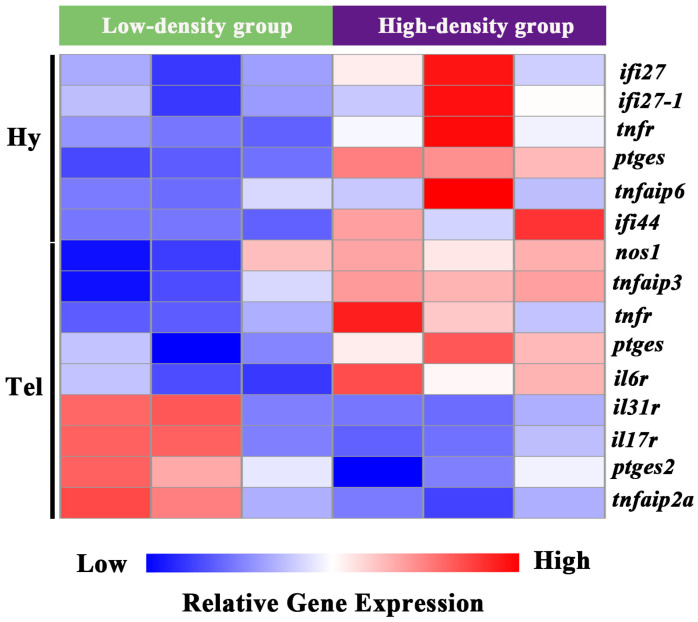
Clustering analysis of DEGs involved in inflammation process in the telencephalon and hypothalamus of largemouth bass after crowding stress. Hy, hypothalamus. Tel, telencephalon. *ifi27*, interferon alpha-inducible protein 27. *ifi27-1*, interferon alpha-inducible protein 27-1. *tnfr*, tumor necrosis factor receptor superfamily member. *ptges*, prostaglandin E synthase. *tnfaip6*, tumor necrosis factor alpha-induced protein 6. *ifi44*, interferon-induced protein 44. *nos1*, nitric oxide synthase 1. *tnfaip3*, tumor necrosis factor alpha-induced protein 3. *il6r*, interleukin-6 receptor. *il31r*, interleukin-31 receptor. *il17r*, interleukin 17 receptor. *ptges2*, prostaglandin E synthase 2. *tnfaip2a*, tumor necrosis factor alpha-induced protein 2a. Relative gene expression levels are represented by a color gradient: red represents gene expression up-regulation, blue represents gene expression down-regulation, and white indicates gene expression with non-significant changes.

Pro-inflammatory-related molecules were specifically screened from the DEGs of the telencephalon and hypothalamus. In the hypothalamus, interferon alpha-inducible protein 27 (*ifi27*, *ifi27-1*), tumor necrosis factor receptor superfamily member (*tnfr*), prostaglandin E synthase (*ptges*), tumor necrosis factor alpha-induced protein 6 (*tnfaip6*), and interferon-induced protein 44 (*ifi44*) were significantly induced. Similarly, in the telencephalon, nitric oxide synthase 1 (*nos1*), tumor necrosis factor alpha-induced protein 3 (*tnfaip3*), *tnfr*, *ptges*, and interleukin-6 receptor (*il6r*) were significantly induced. In contrast, the telencephalon exhibited significant down-regulation (*p* < 0.05) of pro-inflammatory mediators, including interleukin-31 receptor (*il31r*), interleukin-17 receptor (*il17r*), prostaglandin E synthase 2 (*ptges2*), and tumor necrosis factor alpha-induced protein 2a (*tnfaip2a*) (Figure 4).

**Figure 5:**
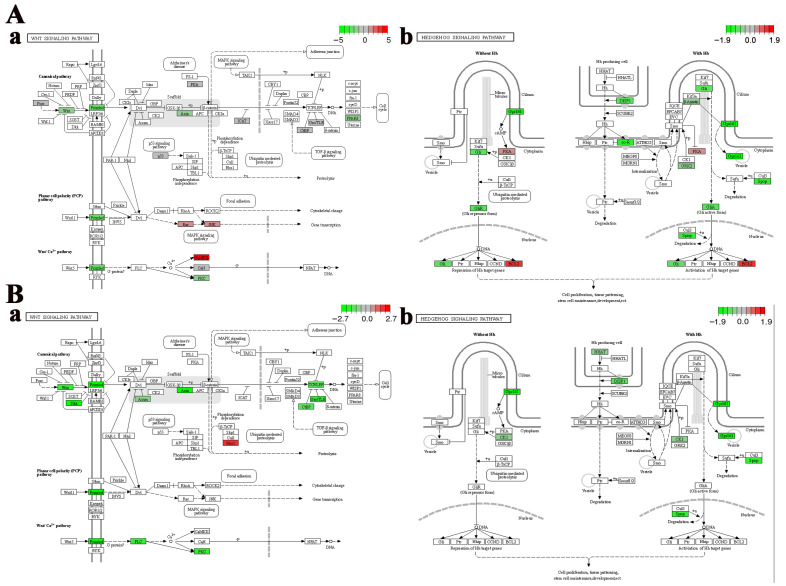
Region-specific dysregulation of Wnt and Hedgehog signaling pathways. (**A**) Telencephalon: (**a**) Wnt, (**b**) Hedgehog. (**B**) Hypothalamus: (**a**) Wnt, (**b**) Hedgehog. Relative gene expression levels of genes in each signaling pathway are represented by a color gradient: red represents gene expression up-regulation, green represents gene expression down-regulation, gray indicates gene expression with non-significant changes, and white denotes genes that are not identified in the transcriptome data.

**Figure 6:**
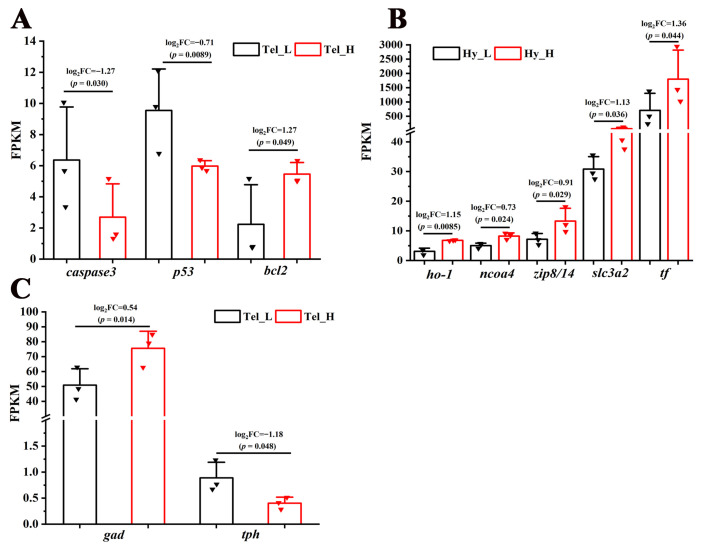
Transcriptional regulation of apoptosis, ferroptosis, and neurotransmitter synthesis processes in response to crowding stress. (**A**) Apoptosis-related genes (*caspase 3*, *p53*, *bcl2*) in the telencephalon; (**B**) ferroptosis-related genes (*ho-1*, *ncoa4*, *zip8/14*, *slc3a2*, *tf*) in the hypothalamus; (**C**) neurotransmitter synthesis-related genes (*gad*, *tph*) in the telencephalon. *Caspase 3*, cysteine-dependent aspartate-specific protease 3. *p53*, tumor protein 53. *bcl2*, B-cell lymphoma-2. *ho-1*, heme oxygenase-1. *ncoa-4*, nuclear receptor coactivator 4. *zip8/14*, metal cation symporter ZIP8. *slc3a2*, solute carrier family 3 member 2. *tf*, transferrin. *gad*, glutamic acid decarboxylase. *tph*, tryptophan hydroxylase. Tel_L, low-density telencephalon. Tel_H, high-density telencephalon. Hy_L, low-density hypothalamus. Hy_H, high-density hypothalamus. The black bars represent the low-density group, while the red bars represent the high-density group. Data are presented as mean ± SD. Statistical significance was determined using DESeq, with *p* < 0.05 considered significant and *p* < 0.01 considered highly significant.

Cell fate-related pathways, including the “Wnt signaling pathway” and the “Hedgehog signaling pathway”, were significantly enriched in the telencephalon and hypothalamus after crowding stress. It was found that most of the genes closely associated with cell growth within these pathways, such as wingless-type MMTV integration site family (*wnt*), frizzled class receptor (*frizzled*), transcription factor 7 (*tcf*), G protein-coupled receptor 161 (*gpr161*), dispatched 1 (*disp1*), and zinc finger protein GLI (*gli*), exhibited down-regulated expression (Figure 5), suggesting that chronic crowding stress inhibits neurogenesis in the telencephalon and hypothalamus. Additionally, three crucial apoptosis-related genes, including *caspase3*, *p53*, and *bcl2*, were identified among the DEGs in the telencephalon after crowding stress. Compared with the low-density group, the expression levels of the apoptosis-promoting genes *caspase3* and *p53* were significantly downregulated in the high-density group, with log_2_Fold Change values of −1.27 and −0.71, respectively (*p* < 0.05). In contrast, the expression level of the apoptosis-inhibiting gene *bcl2* exhibited significant up-regulation in the high-density group (log_2_Fold Change = 1.27, *p* < 0.05) (Figure 6A). Furthermore, ferroptosis-related genes, including *ho-1*, *ncoa4*, *zip8/14*, *slc3a2*, and *tf*, were significantly enriched in the hypothalamus, with log_2_Fold Change values of 1.15, 0.73, 0.91, 1.13, and 1.36, respectively (*p* < 0.05). The coordinated up-regulation of these ferroptosis regulators in the high-density group suggested that ferroptosis was induced in the hypothalamus after crowding stress (Figure 6B).

### 3.5. Impact of Crowding Stress on Neurotransmitter Synthase and Synaptic Plasticity in the Telencephalon and Hypothalamus of Largemouth Bass

To more thoroughly examine crowding stress’s impact on neurochemical signaling, analyses were conducted on the synthases of seven fundamental neurotransmitters (glutamate, γ-aminobutyric acid, glycine, dopamine, serotonin, noradrenaline, and acetylcholine). While only two neurotransmitter synthase genes were enriched among the DEGs in the telencephalon, but not in the hypothalamus, after crowding stress. Compared with the low-density group, the expression level of the GABAergic neuron marker *gad* was significantly higher in the high-density group (log_2_Fold Change = 0.54, *p* < 0.05). In contrast, the expression level of the serotonergic neuron marker *tph* was significantly downregulated in the high-density group (log_2_Fold Change = −1.18, *p* < 0.05) (Figure 6C).

The genes related to synaptic plasticity were also highly enriched in the DEGs from the telencephalon and hypothalamus after crowding stress. Specifically, 20 and 13 genes associated with synaptic plasticity were differently expressed in the telencephalon and hypothalamus, respectively. Among them, twelve genes, including protein kinase C (*prkcg*), voltage-gated calcium channel (*cacna1d*), adenylate cyclase (*adcy2*), ryanodine receptor (*ryr1*, *ryr3*, and *ryr3-1*), glutamate ionotropic receptors (*gria4*, *grik2*, *grin2d*, and *gria4-1*), neurexin (*nrxn3b*), and FHOD protein (*fhod1*), were significantly down-regulated in both the telencephalon and hypothalamus (*p* < 0.05) (Table 3).

### 3.6. Validation of DEGs by qPCR

**Figure 7:**
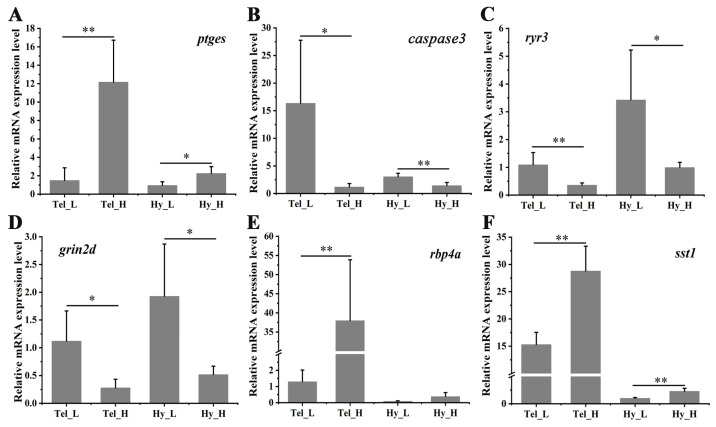
Differential mRNA expression of selected genes in the telencephalon and hypothalamus after crowding stress. (**A**) Relative mRNA expression of prostaglandin E synthase (*ptges*); (**B**) Relative mRNA expression of cysteine-dependent aspartate-specific protease 3 (*caspase 3*); (**C**) Relative mRNA expression of ryanodine receptor 3 (*ryr3*); (**D**) Relative mRNA expression of glutamate receptor ionotropic, NMDA 2D (*grin2d*); (**E**) Relative mRNA expression of retinol-binding protein 4A (*rbp4a*); (**F**) Relative mRNA expression of somatostatin 1 (*sst1*). Tel_L, low-density telencephalon. Tel_H, high-density telencephalon. Hy_L, low-density hypothalamus. Hy_H, high-density hypothalamus. Data are presented as mean ± SD. Statistical significance was determined using SPSS (version 27.0), with *p* < 0.05 considered statistically significant (*), and *p* < 0.01 considered highly statistically significant (**).

To assess the accuracy of RNA-seq, a qPCR was conducted on six representative genes. As shown in Figure 7, the inflammation-related gene (*ptges*) exhibited significant up-regulation in both the telencephalon and hypothalamus after four weeks of crowding stress. In contrast, the apoptosis-related gene (*caspase3*) and the synaptic plasticity-related genes (*ryr3*, *grin2d*) were significantly down-regulated in both brain regions following crowding stress. The retinol-binding protein 4A gene was highly induced in the telencephalon (*p* < 0.01), while somatostatin 1 showed significant induction in both the telencephalon and hypothalamus (*p* < 0.01) in the high-density group compared to the low-density group. Collectively, these findings indicate strong concordance between the qPCR and RNA-seq expression profiles, validating the reliability of the RNA-seq data.

## 4. Discussion

Neuroinflammation serves as a hallmark of chronic stress, well-documented in rodents and zebrafish models [37]. For instance, it was found that chronic stress induced neuroinflammatory changes in mice, which were regulated by type 1 interferon [38]. Similarly, five weeks of chronic stress significantly elevated the expression of pro-inflammatory marker genes in the central nervous system (CNS) of adult zebrafish [37]. However, research on chronic stress-induced neuroinflammation in cultured fish species remains limited. In the present study, inflammation-related molecules (*ifi27*, *tnfr*, *ptges*, *il6r*, and *il17r*) have been found to be highly expressed in the telencephalon and hypothalamus of crowding-stressed largemouth bass, indicating crowding stress-induced neuroinflammation. Accumulating evidence from both mammalian and teleost models demonstrates that chronic stress compromises blood–brain barrier (BBB) integrity, promoting the translocation of peripheral cytokines and immune cells into the central nervous system and consequently exacerbating neuroinflammatory responses [39]. For instance, in zebrafish, increased BBB permeability was observed after five weeks of chronic stress, which was inferred to promote neuroinflammation through the entry of peripheral cytokines into the brain [37]. Similarly, cold stress disrupted the BBB in leopard coral grouper (*Plectropomus leopardus*), accompanied by pronounced neuroinflammation [40]. Our latest findings further confirm that long-term crowding stress indeed enhances BBB permeability in the telencephalon of the largemouth bass (unpublished). These results collectively suggest that neuroinflammation may be induced by the infiltration of inflammatory cytokines from the periphery into the brain through a compromised BBB. Notably, significant enrichment of the “Tight junction” pathway, accompanied by an upregulated expression of the genes encoding tight junction-associated proteins (*Claudin*, *Occludin*, and *Tight junction-associated protein*), was observed in the present study. While crowding stress may disrupt intercellular junctions and further increase BBB permeability [41], the observed up-regulation of these genes likely represents a compensatory mechanism to restore barrier integrity during chronic stress exposure.

In contrast to the limited neurogenesis capacity of adult mammalian brains, teleost brains maintain lifelong neurogenesis, a process highly sensitive to environmental stressors [42]. For instance, it was reported that predator stress significantly inhibits cell proliferation in the forebrain of electric fish, *Brachyhypopomus occidentalis* [43]. Similarly, restraint stress altered the neurogenesis of rainbow trout (*Oncorhynchus mykiss*), and the effects depended on the duration of stress exposure. Specifically, acute stress (2 h) upregulated the expression of the neurogenic markers of the proliferating cell nuclear antigen (PCNA) and brain-derived neurotrophic factor (BDNF) in the brain regions of the telencephalon, hypothalamus, and optic tectum. In contrast, chronic stress (5 days) had suppressive effects on the hypothalamus, cerebellum, and optic tectum [44]. Consistently, 10 days of chronic unpredictable stress attenuated neurogenesis in the telencephalon of the zebrafish [45]. In our present study, although no significant changes in the expression levels of BDNF and PCNA genes have been observed in the telencephalon and hypothalamus after stress, a pronounced down-regulation of the key genes within the Wnt and Hedgehog pathways was observed, which are crucial regulators of neural cell maintenance and differentiation. These results collectively suggest that crowding stress inhibits neurogenesis in the telencephalon and hypothalamus of largemouth bass. Synaptic plasticity represents another adaptation mechanism by which fish respond to environmental stress through physiological and behavioral regulation [44]. Our transcriptome analysis revealed a significant down-regulation of key synaptic plasticity-related genes in the telencephalon and hypothalamus of crowding-stressed largemouth bass, including ryanodine receptor, glutamate receptor ionotropic, and formin homology 2 domain-containing 1 [46,47,48]. This finding suggests that long-term crowding stress suppresses synaptic plasticity in these two brain regions. Additionally, the genes of retinol-binding protein 4A (RBP4A) and cathepsin D, which were among the top 10 upregulated genes in the telencephalon, were also closely linked to neurogenesis and synaptic plasticity [49,50]. Under stressful conditions, neurogenesis and synaptic plasticity in the brain serve as the foundation for behavioral adaptation in fish [51]. Reduced neurogenesis and impaired synaptic plasticity can lead to behavioral alterations, including diminished spatial learning and memory, reduced locomotor flexibility, decreased exploratory behavior, and increased anxiety- and depression-like behaviors, as well as heightened aggression [52,53,54]. These behavioral changes are consistent with the previous observations of abnormal behaviors induced by long-term crowding in fish [3]. Collectively, these results indicate that dysregulated neurogenesis and synaptic plasticity in the brain regions of largemouth bass, triggered by crowding stress, may be a key factor underlying their behavioral abnormalities.

Apoptosis serves as another critical cell fate process for maintaining neuronal populations and functionality [44]. Our transcriptomic analysis revealed a significant down-regulation of key pro-apoptotic genes (*caspase 3* and *p53*) coupled with an up-regulation of the anti-apoptotic gene *bcl2* in the telencephalon of crowding-stressed largemouth bass, indicating that crowding stress suppresses apoptosis in telencephalon cells. This observation aligns with a report showing that three weeks of chronic unpredictable stress decreased apoptosis in the hippocampal dentate gyrus of rats [55]. However, it contrasts with studies that five weeks of chronic unpredictable stress increased neuronal apoptosis in the cerebral cortex [56]. These discrepancies may arise from differences in stress duration. It is speculated that the 3–4 weeks of stress in our study were moderate, triggering a self-protective mechanism in which the brain compensates for reduced neurogenesis by inhibiting apoptosis to maintain neuronal numbers. In contrast to the telencephalon, ferroptosis rather than apoptosis was induced in the hypothalamus of largemouth bass following stress. Ferroptosis, as a novel iron-dependent programmed cell death, is initiated through excessive ROS production and subsequent lipid peroxidation [57]. A recent study demonstrated that two months of chronic, unpredictable mild stress significantly activated ferroptosis in the mouse hippocampus [58]. Similarly, in teleosts, chronic mercury exposure induced neuronal ferroptosis in common carp (*Cyprinus carpio*). Furthermore, in Nile tilapia (*Oreochromis niloticus*), temperature shock exposure markedly increased brain lipid peroxidation. However, the specific brain regions undergoing ferroptosis remain uncharacterized in teleosts [59,60]. Collectively, our results suggest that crowding stress triggers distinct modes of cell death in the telencephalon and hypothalamus, exhibiting regional heterogeneity of crowding stress. The regional heterogeneity of cell death patterns in the brain may be attributed to the distinct functional characteristics of different brain regions. The hypothalamus, as the primary neuroendocrine regulator during stress exposure, requires high energy expenditure upon activation. This elevated metabolic demand results in increased reactive oxygen species (ROS) production and subsequent lipid peroxidation. Furthermore, the antioxidant defense system of the hypothalamus, which primarily relies on glutathione (GSH), is less effective compared to that of the telencephalon [61]. Therefore, it can be inferred that the hypothalamus is more susceptible to ferroptosis under stress conditions. In contrast, the telencephalon is mainly involved in learning, memory, and synaptic plasticity. These processes are closely related to the apoptosis process [18]. Consequently, apoptosis inhibition is predominantly observed in the telencephalon. However, further research is needed to determine whether the apoptotic process is promoted with prolonged exposure to crowding stress.

Neurotransmitters, acting as crucial messengers between neurons, play a pivotal role in multiple physiological processes, including emotion, memory, motivation, reward, and food intake [62]. The telencephalon of fish is a crucial brain region associated with emotion, learning, and memory, and a region analogous to the amygdala and hippocampus in mammals, which are also involved in these functions [26,44]. Numerous reports have suggested that chronic crowding stress alters telencephalic neurotransmitter dynamics in teleosts. For example, in *O. mykiss*, 28 days of crowding stress significantly decreased serotonin content in the telencephalon [16]. Consistently, in our present study, the expression of the serotonin synthase (tryptophan hydroxylase) gene in the telencephalon of largemouth bass was significantly down-regulated in the high-density group, suggesting that serotonin (5-HT) content decreased after crowding stress, which might be directly related to anxiety- or depression- like emotions induced by crowding stress. Notably, we observed significant up-regulation of γ-aminobutyric acid (GABA) synthase gene expression in the telencephalon of largemouth bass after crowding stress, indicating enhanced GABAergic neurotransmission following four weeks of density exposure. Our result is discrepant with reports in mammals that chronic stress causes deficits in the GABAergic inhibitory neurotransmitter within the prefrontal cortex [63]. This discrepancy might be attributed to the moderate stress level in our study, which enabled the body’s compensatory responses to GABA. Furthermore, numerous reports have suggested that neurotransmitters like 5-HT and GABA are closely related to neurogenesis and synaptic plasticity [64,65], which warrants further exploration in fish under crowding stress.

The growth process of aquatic animals is regulated by the neuroendocrine system, with the hypothalamus being a critical brain region involved in growth regulation [66,67]. The hypothalamus directly influences fish growth by modulating neuroendocrine hormones such as the growth hormone (GH), somatostatin, and thyroid hormones [68]. Our present study found that these growth-related hormones are significantly enriched in the DEGs in the hypothalamus. For example, thyroid-stimulating hormone subunit beta (*tshb*) and somatostatin 1 (*sst1*) were enriched in the top 10 down-regulated and top 10 up-regulated genes in the hypothalamus, respectively. The genes of insulin-like growth factor 2 mRNA-binding protein (*igf2bp*) and thyroid hormone receptor-associated protein (*thrap*) were also significantly enriched in the DEGs of the hypothalamus, exhibiting significant down-regulation after crowding stress. According to our previous study, growth retardation of adult largemouth bass was observed after four weeks of crowding stress [20]. Combining this with the present study, it is suggested that the down-regulation of growth-related components in the hypothalamus may be the underlying cause of growth suppression in largemouth bass under chronic crowding stress. These results align with previous reports in flounder [69], sturgeon [70], and rainbow trout [14], where long-term crowding stress-induced growth retardation was attributed to the negative regulation of the GH-IGF (growth hormone–insulin-like growth factor) axis. Nevertheless, the precise molecular mechanisms underlying the down-regulation of this axis have not yet been fully elucidated. Combining the up-regulation of somatostatin in the present study, which primarily functions to inhibit GH release, it is inferred that somatostatin in the hypothalamus might regulate the GH-IGF axis to contribute to growth suppression under crowding stress. Although the representative genes have been validated by qPCR in our study, other functional verifications have not been carried out to interpret the transcriptome changes. This is a limitation of our study, which will be explored in our future research.

## 5. Conclusions

**Figure 8:**
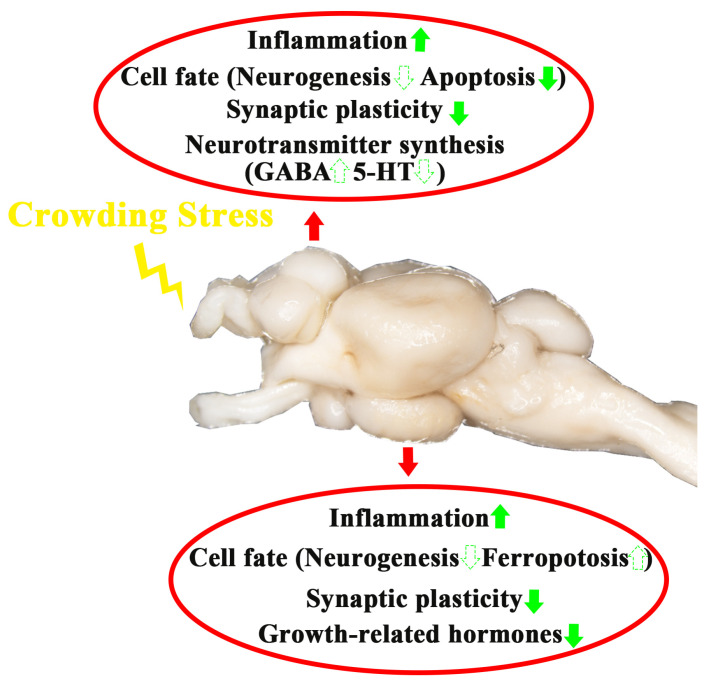
A schematic model depicting the influence of crowding stress on the telencephalon and hypothalamus of largemouth bass. The green upward arrow indicates up-regulation of gene expression. The green downward arrow indicates down-regulation of gene expression. The solid arrows represent genes in pathways that have been validated by qPCR in the present study, while the dashed arrows represent speculative pathways.

In summary, after four weeks of crowding stress, the following effects were observed in the telencephalon and hypothalamus (Figure 8):

- Neuroinflammatory induction;
- Region-specific alterations in cell death pathways:
  - Apoptosis down-regulation in the telencephalon;
  - Ferroptosis induction in the hypothalamus.
- Synaptic plasticity attenuation;
- Neurotransmitter/hormone dysregulation:
  - γ-aminobutyric acid and serotonin synthesis changed in the telencephalon;
  - Thyroid-stimulating hormone and somatostatin altered in the hypothalamus.

Collectively, these findings offer novel insights into the brain response mechanisms underlying chronic crowding stress in fish. These results underscore the potential of targeting anti-inflammatory processes, ferroptosis inhibition, or neural plasticity enhancement to mitigate the detrimental effects induced by crowding and improve welfare in intensive aquaculture systems through environmental enrichment, dietary interventions, or selective breeding.

## Figures and Tables

**Table 1:** Top 10 up-regulated and down-regulated genes in the telencephalon of largemouth bass after four weeks of crowding stress.

Gene Name	Description	Brief Function	H_Tel_FPKM	L_Tel_FPKM	*p* Value	Log_2_Fold Change
*cndp2*	cytosolic non-specific dipeptidase	Protease, Neuroprotection, Inflammation, Oxidative stress	52.65	2.00	0.0064	4.68
*actr1b*	actin-related protein 1B	Neural development, axonal growth, synaptic plasticity	19.03	2.74	2.85 × 10^−11^	2.77
*rbp4a*	retinol-binding protein 4A	Transport of vitamin A, neurogenesis, synaptic plasticity, inflammation	23.21	3.45	3.46 × 10^−6^	2.72
*hagh*	hydroxyacylglutathione hydrolase	Cellular detoxification, antioxidant defense, glucose metabolic homeostasis	5.57	1.32	0.0055	2.06
*cbln1*	cerebellin-1	Synaptogenesis, neurotransmitter transmission, synaptic plasticity	15.28	3.70	0.00013	2.03
*dgkh*	diacylglycerol kinase	Lipid metabolism, synaptic plasticity	11.71	2.84	0.00062	2.02
*tm9sf2*	transmembrane 9 superfamily member 2	Endosomes and lysosomes activities, antiviral responses	6.15	1.53	6.44 × 10^−5^	1.98
*ctsd*	cathepsin D	Protein degradation, apoptosis, inflammation, neurogenesis, synaptic plasticity	25.51	6.83	2.67 × 10^−5^	1.86
*praf2*	PRA1 domain family member 2	Vesicular transport, neurotransmitter transport, apoptosis	13.21	3.59	0.016	1.86
*rtn4rl2*	reticulon-4 receptor-like 2	Synaptic growth and plasticity, apoptosis, endoplasmic reticulum stress	27.51	7.73	1.67 × 10^−7^	1.81
*taf9*	TAF9 RNA polymerase II	Transcription initiation process, apoptosis, neuronal proliferation and differentiation, lipid metabolism	13.02	33.37	4.55 × 10^−6^	−1.39
*dedd2*	DNA-binding death effector domain-containing protein 2	Apoptosis	14.06	37.58	0.0030	−1.46
*p4htm*	prolyl 4-hydroxylase transmembrane	Hypoxia response, inflammation, blood–brain barrier permeability, calcium signaling	8.31	22.57	0.00090	−1.48
*rpl7a*	ribosomal protein L7a	Protein translation, axonal regeneration, synaptic plasticity	5.14	14.27	0.011	−1.51
*rab7*	Ras-related protein 7	Membrane transport, autophagy	5.98	16.66	0.00014	−1.52
*cfi*	complement factor I	Immune defense, neuroinflammation, neuroprotective	6.40	18.48	0.0025	−1.57
*oc90*	otoconin 90	Vestibular balance	10.14	32.56	1.85 × 10^−5^	−1.72
*styk1*	tyrosine-protein kinase	Cell proliferation and differentiation, neurogenesis	6.78	21.88	0.00019	−1.73
*prkcg*	protein kinase C gamma	Synaptic plasticity, neuroprotective	8.89	29.59	0.016	−1.78
*trappc1*	trafficking protein particle complex subunit 1	Vesicular transport	6.00	20.95	0.0027	−1.84

**Table 2:** Top 10 up-regulated and down-regulated genes in the hypothalamus of largemouth bass after four weeks of crowding stress.

Gene Name	Description	Brief Function	H_Hy_FPKM	L_Hy_FPKM	*p* Value	Log_2_Fold Change
*ddta*	D-dopachrome decarboxylase-A-like	Melanin biosynthesis, immune function	5.97	0.07	4.15 × 10^−8^	6.36
*chchd10*	coiled-coil-helix-coiled-coil-helix domain-containing protein 10	Energy metabolism, oxidative stress, neurodegeneration processes	20.04	0.45	0.00028	5.54
*iaip27*	interferon alpha-inducible protein 27	Antiviral responses, apoptosis, inflammation	27.60	1.94	0.00035	3.88
*mdh1*	malate dehydrogenase	Energy metabolism	44.18	3.38	2.42 × 10^−9^	3.71
*atp5*	ATP synthase subunit	ATP biosynthesis, oxidative stress, neurodegeneration	13.15	1.05	0.0013	3.64
*pnrc2*	proline-rich nuclear receptor coactivator 2	Hormone synthesis, fat metabolism	24.89	2.30	0.0008	3.48
*sst1*	somatostatin 1	Growth hormone release, neuronal activity, synaptic plasticity, memory formation	22.42	2.66	0.0029	3.11
*ccmc5*	C-C motif chemokine 5	Inflammation	9.28	1.23	0.011	2.95
*icl*	ictacalcin-like	Calcium homeostasis	17.36	2.41	0.00048	2.87
*iaip27-1*	interferon alpha-inducible protein 27	Antiviral responses, apoptosis, inflammation	32.27	5.32	0.0011	2.64
*neub1*	neurabin-1	Synaptic plasticity, synaptic formation	2.43	9.80	0.002	−2.00
*gucy1b2*	guanylate cyclase soluble subunit beta-2	NO receptor, synaptic plasticity	2.18	9.15	0.00012	−2.06
*mycbpap*	MYCBP-associated protein	Cell proliferation, synaptic remodeling	3.31	14.48	0.0015	−2.12
*kbtbd3*	kelch repeat and BTB (POZ) domain containing 3	Ubiquitination	4.01	18.03	0.00081	−2.16
*tshb*	Thyroid-stimulating hormone subunit beta	Growth, reproduction	2.02	9.67	0.034	−2.23
*alox15b*	polyunsaturated fatty acid lipoxygenase ALOX15B	Inflammation	1.46	7.33	0.0017	−2.32
*trio*	Triple-functional domain protein	Neurogenesis, neuronal migration, synaptic plasticity	0.73	5.55	0.0011	−2.92
*pgrs16*	PE-PGRS family protein	*Mycobacterium tuberculosis* surface antigens	0.70	8.52	3.96 × 10^−9^	−3.59
*gtf2a2*	transcription initiation factor IIA subunit 2	Transcription initiation process	0.32	8.63	2.29 × 10^−9^	−4.77
*slc8a1*	NAC1_CANLF sodium/calcium exchanger 1	Calcium homeostasis	0.08	6.30	5.75 × 10^−12^	−6.28

**Table 3:** Impact of crowding stress on genes related to synaptic plasticity in the telencephalon and hypothalamus of largemouth bass.

Gene Name	Description	Tel	Hy
		Log_2_Fold Change (*p* Value)	Log_2_Fold Change (*p* Value)
*prkcg*	protein kinase C	−1.78 (0.016)	−1.99 (0.0025)
*cacna1d*	voltage-gated calcium channel subunit alpha	−0.88 (0.019)	−1.52 (0.075)
*adcy2*	adenylate cyclase type 2	−1.03 (0.025)	−1.08 (0.031)
*ryr1*	ryanodine receptor 1	−0.66 (0.038)	−0.94 (0.032)
*ryr3*	ryanodine receptor 3	−0.75 (0.012)	−1.04 (0.027)
*ryr3-1*	ryanodine receptor 3-1	−0.89 (0.0099)	−1.45 (0.0040)
*grm4*	metabotropic glutamate receptor 4	1.38 (0.028)	-
*grin2a*	glutamate receptor ionotropic, NMDA 2A	0.72 (0.021)	-
*grik3*	glutamate receptor ionotropic, kainate 3	0.67 (0.0037)	-
*grm5*	metabotropic glutamate receptor 5	0.31 (0.017)	-
*gria3*	glutamate receptor ionotropic, AMPA 3	−0.74 (0.046)	-
*gria4*	glutamate receptor ionotropic, AMPA 4	−0.87 (0.010)	−1.16 (0.017)
*grik2*	glutamate receptor ionotropic, kainate 2	−0.93 (0.011)	−1.64 (0.034)
*grin2d*	glutamate receptor ionotropic, NMDA 2D	−0.94 (0.0065)	−1.16 (0.019)
*gria4-1*	glutamate receptor ionotropic, AMPA 4-1	−1.01 (0.0056)	−1.25 (0.026)
*grm7*	metabotropic glutamate receptor 7	−1.16 (0.027)	-
*grin2b*	glutamate receptor ionotropic, NMDA 2B	-	−0.80 (0.037)
*lrrtm4*	leucine-rich repeat transmembrane neuronal 4	1.10 (0.015)	-
*nrxn3b*	neurexin 3b	−0.63 (0.047)	−0.94 (0.033)
*fhod1*	formin homology 2 domain containing 1	−1.03 (0.00097)	−1.55 (0.000032)
*bmp6*	bone morphogenetic protein 6-like	−1.49 (0.0062)	-

## Data Availability

The raw sequencing data are publicly available in the Genome Sequence Archive (GSA) at the China National Center for Bioinformation (CNCB). The accession number is GSA: CRA024519.

## Abbreviations

The following abbreviations are used in this manuscript:

Tel	Telencephalon
Hy	Hypothalamus
FPKM	Fragments Per Kilobase per Million
GABA	γ-aminobutyric acid
5-HT	Serotonin

## Appendix A

**Table A1:** Summary of the mRNA-seq data.

Sample	Raw Reads (10^6^)	Clean Reads (10^6^)	Mapping Reads (10^6^)	Mapping Rate (%)	Unique Mapping Reads (10^6^)	Unique Mapping Rate (%)
L_Tel_1	46.56	44.60	42.62	95.57	40.54	90.91
L_Tel_2	45.31	43.08	41.18	95.59	39.20	91.00
L_Tel_3	45.63	43.36	41.54	95.80	39.31	90.64
H_Tel_1	45.57	43.34	41.75	96.32	39.56	91.28
H_Tel_2	45.55	43.17	41.45	96.01	39.22	90.83
H_Tel_3	45.12	42.92	41.10	95.76	38.82	90.45
L_Hy_1	44.58	42.63	40.90	95.94	38.95	91.37
L_Hy_2	44.27	42.37	40.77	96.23	38.82	91.62
L_Hy_3	43.21	41.43	39.99	96.53	37.92	91.52
H_Hy_1	46.67	43.60	41.99	96.33	39.77	91.21
H_Hy_2	45.23	42.75	40.61	95.00	38.62	90.34
H_Hy_3	46.49	44.43	42.94	96.64	40.69	91.59

Note: L_Tel, low-density telencephalon. H_Tel, high-density telencephalon. L_Hy, low-density hypothalamus. H_Hy, high-density hypothalamus.

**Table A2:** Primers for qPCR.

Gene Name	Forward Primer	Reverse Primer
*ptges*	GCTGAGACACGGAGGTTTAC	ACAGGCAGTGAAGGTCCAATC
*caspase3*	GAAGAGCAAACAGAGAGGATT	TTGGAGGCAGATTCAAAGTG
*ryr3*	AACTGATGACGAGGTGGTGC	GGTCTGGAGGAACATACTTAGC
*grin2d*	GGTATTTCGGCAACATCACA	TCCCACCAACCTACATCTTC
*rbp4a*	TAGCAAAGAAGGACCCAGAG	GCGTAGTTGTCGTAGTCAGTG
*sst1*	CTCACTCACAGCCTCCATCAGC	CCCAGAGCCTCGTTCTCCACC
*β-actin*	AAAGGGAAATCGTGCGTGAC	AAGGAAGGCTGGAAGAGGG
